# ECG Signal Denoising and Features Extraction Using Unbiased FIR Smoothing

**DOI:** 10.1155/2019/2608547

**Published:** 2019-02-20

**Authors:** Carlos Lastre-Domínguez, Yuriy S. Shmaliy, Oscar Ibarra-Manzano, Jorge Munoz-Minjares, Luis J. Morales-Mendoza

**Affiliations:** ^1^Universidad de Guanajuato, Department of Electronics Engineering, Salamanca 36885, Gto., Mexico; ^2^Universidad Veracruzana, Department of Electronics Engineering, Poza Rica 93390 Ver., Mexico

## Abstract

Methods of the electrocardiography (ECG) signal features extraction are required to detect heart abnormalities and different kinds of diseases. However, different artefacts and measurement noise often hinder providing accurate features extraction. One of the standard techniques developed for ECG signals employs linear prediction. Referring to the fact that prediction is not required for ECG signal processing, smoothing can be more efficient. In this paper, we employ the *p*-shift unbiased finite impulse response (UFIR) filter, which becomes smooth by *p* < 0. We develop this filter to have an adaptive averaging horizon: optimal for slow ECG behaviours and minimal for fast excursions. It is shown that the adaptive UFIR algorithm developed in such a way provides better denoising and suboptimal features extraction in terms of the output signal-noise ratio (SNR). The algorithm is developed to detect durations and amplitudes of the P-wave, QRS-complex, and T-wave in the standard ECG signal map. Better performance of the algorithm designed is demonstrated in a comparison with the standard linear predictor, UFIR filter, and UFIR predictive filter based on real ECG data associated with normal heartbeats.

## 1. Introduction

The electrocardiography (ECG) signals play a key role in diagnosing diverse kinds of heart diseases. Because the pulses produced by heart may have subtle differences from each other and noise affects the decision accuracy, the ECG is commonly organized using precise electronic equipment [1]. Accurate measurements are especially required when data are used to extract features of ECG signals and make decisions about different kinds of heart diseases employing special software. However, even very precise measurements are typically contaminated by artefacts and noise. Artefacts may result from a variety of internal and external causes, such as the Parkinsonian muscle tremors drying electrode gel. Different kinds of noises may contaminate the ECG signal during its acquisition and transmission, such as the high frequency noise (electromyogram noise, additive white Gaussian noise, and power line interference) and low frequency noise (baseline wandering). Because noise may lead to wrong interpretation, ECG signal denoising is required. Therefore, significant attention has been paid during the last decades to develop mathematical methods and computation algorithms to extract the ECG features from regular (noisy) data with an accuracy sufficient for medical needs [2–12].

The Fourier transform-based approach has been developed in [13] to extract ECG signal features in the frequency domain. But, this method omits the time resolution, which affects the estimation accuracy. This issue has been circumvented in some other works by providing the time-frequency analysis without significantly affecting the resolution. In [14–17], the wavelet transform-based algorithms were developed to find applications in some medical areas. In the wavelet domain, a compromise between the frequency and time resolutions is achieved easier and one can select a proper wavelet to provide a reasonable accuracy. However, a choice of an optimal wavelet is still challenging [18] and the approach has low efficiency in smoothing ECG signals. Other algorithms tested for such needs include the principal component analysis (PCA) [19], linear discriminant analysis (LDA) [20], independent component analysis (ICA) [21], support vector machine [22], and neural networks [23].

One of the widely recognized approaches proposed in [24] provides noise reduction and features extraction from ECG data by employing linear prediction based on the theory developed in [25]. The approach suggests that all main features of ECG signals can be saved and gained using a one-step linear predictor. Accordingly, features extraction in the QRS complex (region of fast ECG excursions) is provided from an analysis of residual errors between the data and estimates. The approach has manifested itself as useful in the detection of arrhythmias. In other works employing one-step prediction [26, 27], automatic classification of the ECG cardiac abnormalities is provided using Gaussian mixtures. Later, the prediction-based approach has been recognized as one of the standard techniques suitable for ECG signals [28].

It has to be remarked now that, from the standpoint of optimal filtering,* prediction* is less accurate in noise reduction than* filtering* and much less accurate than* smoothing*. On the other hand, the ECG signal processing problems do not imply predicting future values and smoothing with some time-lag may be a better choice for cardiac analysis. A classic example is the Savitzky-Golay filter (smoother) [29], which has found wide applications in diverse areas [30–35].

An optimal approach to provide smoothing and state estimation in linear models has been proposed in [36] to minimize the mean square error (MSE). A solution was found on a horizon [*m* − *p*, *n* − *p*] of *N* data points, where *n* corresponds to a fixed discrete point of the ECG signal, *m* = *n* − *N* + 1, and *p* is a discrete shift. The derived optimal FIR (OFIR) filter becomes smoothing with lag *q* = −*p* by *p* < 0, provides filtering with *p* = 0, and becomes *p*-step predictive when *p* > 0. However, the *p*-shift OFIR filter requires information about noise, which is not completely available for ECG signals.

A special case of the *p*-shift OFIR filter is the *p*-shift unbiased FIR (UFIR) filter [36–39], which completely ignores zero mean noise and is thus more suitable for ECG signals. As being more general, the *p*-shift UFIR filter generalizes the Savitzky-Golay filter by *p* = −(*N* − 1)/2 and linear predictor with *p* > 0. Although such a filter does not require the noise statistics except for the zero mean assumptions, it provides nice near optimal estimates if *N* is set optimally as *N*_opt_ by minimizing the MSE [36].

In this paper, we develop an adaptive-horizon UFIR smoothing filtering algorithm for denoising ECG signals and features extraction. We also investigate the trade-off between the UFIR smoothing filter, UFIR filter, and UFIR predictive filter and compare them to the standard linear predictor suggested in [24]. We base our investigations on the MIT-BIH Arrhythmia Database available for free use from [40, 41]. Focused on the design of efficient algorithms, in this paper we limit our investigations by data associated with normal heartbeats and postpone an analysis of different kinds of heart diseases to future investigations. The rest of the paper is organized as follows. In Section 2, we describe the database, theory of the algorithms proposed, and validation. The experimental results are showed in Section 3, where we provide a comparison between the UFIR, UFIR smoothing, and UFIR predictive filtering algorithms. A discussion of the results is provided in Section 4 and generalizations with concluding remarks are given in Section 5.

## 2. Material and Methods

### 2.1. Materials

This work employs the MIT-BIH Arrhythmia Database as a benchmark. This database contains 48 ECG recordings applying two leads (e.g., MLII, V1), obtained from 47 subjects studied. The recordings have a sampled frequency 360Hz per channel with 11-bit resolution over a 10 mV range. In general, the lead most common in this database is MLII where the morphology of signal ECG is seen clearly. For testing process the MLII lead and sinusal normal rhythm are considered. Moreover, an additional test with ECG synthetic data is provided by specialized software of MATLAB designed by Karthik Raviprakash. The simulated ECG signals are based on principle of Fourier series. Here, the signal is corrupted by different levels of Gaussian white noise. Below is a brief description of the characteristics of the ECG signal.

#### 2.1.1. ECG Signal

**Figure 1:**
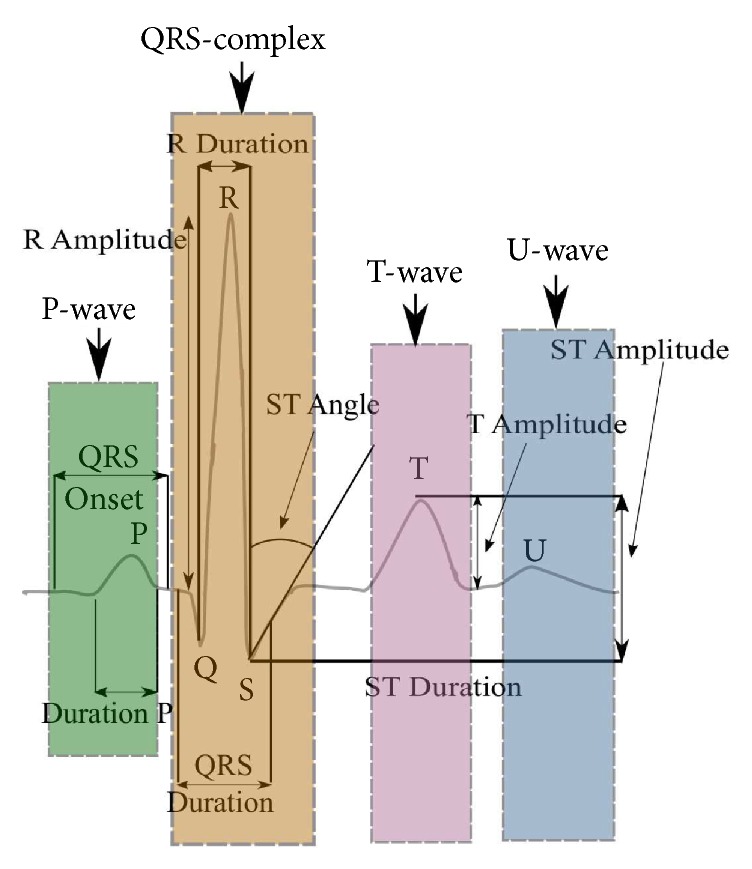
The heartbeat pulse model represented with features (amplitudes and durations) of the P-wave, QRS complex, T-wave, U-wave, and ST angle.

The morphology of heartbeat is fundamental for extracting features of ECG signals, which are quasiperiodic as sketched in Figure 1. The heartbeat pulse can be represented with four fundamental features: P-wave (left slow excursion), QRS-complex (central fast excursion), T-wave (first right slow excursion), and U-wave (second right slow excursion).

Several problems arise while processing ECG signals shown in Figure 1:

- Measurement data are commonly contaminated by noise, which may not be Gaussian and white.
- Standard features depicted in Figure 1 must be estimated with highest accuracy to avoid medical mistakes.
- The ground truth (reference model) is not available to tune an estimator optimally.

Under such conditions, two approaches relying on accurate identification of heartbeat pulses are commonly considered to extract ECG signal features:* fiducial* and* nonfiducial*. The fiducial approach refers to the characteristics such as amplitude and heart rate, which are related to the duration, amplitude, and wave shape [44–49]. The nonfiducial approach refers to quasiperiodicity of ECG signals [28] and all features are separated into three main categories based on autocorrelation, phase-space, and frequency-domain analysis.

### 2.2. Methods

#### 2.2.1. *p*-Shift UFIR Smoothing Filtering

Let us suppose that the ECG signal *x*_*n*_ (Figure 1) is contaminated by zero mean additive noise *v*_*n*_ with unknown statistics. Then measurement *s*_*n*_ of *x*_*n*_ can be represented in discrete-time index *n* as an additive sum of

(1) $$\begin{array}{c} s_{n}=x_{n}+v_{n}. \end{array}$$

In view of the fact that noise *v*_*n*_ in (1) may not be white Gaussian and its statistics are commonly not well-known, the best way to avoid large estimation errors is using filters that do not require information about the statistics of noise. The *p*-shift UFIR filter, which completely ignores noise and the initial conditions, can thus be considered as a good candidate.

On a finite horizon [*m* − *p*, *n* − *p*] of *N* points, the ECG signal can be represented with a degree polynomial and the *p*-shift UFIR filter [37] applied to remove noise. In accordance with [37], the UFIR estimate ${\hat{x}}_{n|n-p}$ of *x*_*n*_ via data *s*_*n*_ taken from [*m* − *p*, *n* − *p*] can be found in the convolution-based form of

(2a) x^n ∣ n−p=∑i=pN−1+phlipsn−i

(2b) =WlTpSNp,

where *h*_*ln*_(*p*)≜*h*_*ln*_(*N*, *p*) is the {*N*, *p*}-variant impulse response of the *l*-degree UFIR filter, the extended measurement vector **S**_*N*_ is

(3) $$\begin{array}{c} S_{N}\left(\;p\;\right)={\left[\;s_{n-p}\;\;s_{n-1-p}\cdots s_{m-p}\;\right]}^{T}, \end{array}$$

and the filter gain matrix is given by

(4) $$\begin{array}{c} W_{l}^{T}\left(\;p\;\right)=\left[\;h_{lp}\left(\;p\;\right)\;h_{l\left(1+p\right)}\left(p\right)\cdots h_{l\left(N-1+p\right)}\left(p\right)\;\right]. \end{array}$$

To satisfy the unbiasedness condition

(5) $$\begin{array}{c} E\left\{\;{\hat{x}}_{n\;|\;n-p}\;\right\}=E\left\{\;x_{n}\;\right\}, \end{array}$$

where *E*{*z*} means an average of *z*, then *h*_*ln*_(*p*) can be represented as [37, 50]

(6) $$\begin{array}{c} h_{li}\left(\;p\;\right)=\overset{l}{\underset{j=0}{\sum }}a_{jl}\left(\;p\;\right)i^{j}, \end{array}$$

where *i* ∈ [*p*, *N* − 1 + *p*] and the coefficients *a*_*jl*_(*p*) are defined by [37]

(7) $$\begin{array}{c} a_{jl}\left(\;p\;\right)={\left(\;-1\;\right)}^{j}\frac{M_{\left(j+1\right)1}\left(p\right)}{\left|D\left(p\right)\right|}. \end{array}$$

Here, |**D**(*p*)| is the determinant of matrix **D**(*p*) = **V**^*T*^(*p*)**V**(*p*), where **V**(*p*) is the *N* × (*l* + 1) Vandermonde matrix,

(8) Vp=1pp2⋯pl11+p1+p2⋯1+pl12+p2+p2⋯2+pl⋮⋮⋮⋱⋮1N−1+pN−1+p2⋯N−1+pl

and *M*_(*j*+1)1_(*p*) is the minor of **D**(*p*). Function *h*_*li*_(*N*, *p*) has the following fundamental properties [37, 50]:

(9) hliN,p=nontrivial,p⩽i⩽N−1+p0,otherwise,

(10) ∑i=pN−1+phliN,p=1,

(11) ∑i=pN−1+phliN,piu=0,1≤u≤l.

For low-degrees, *l* = 1 and *l* = 2, one can find *h*_*li*_(*N*, *p*) in Appendix A. For higher degrees, *h*_*li*_(*N*, 0) can be computed using a recurrence relation [51, 52] and then *h*_*li*_(*N*, *p*) is obtained by a projection. Of importance is that the UFIR estimate (2b) does not require the noise statistics and initial values. The zero mean noise *v*_*n*_ is allowed to have any distribution and covariance [53, 54] that is a fundamental difference with optimal estimates.

#### 2.2.2. ECG Signal Denoising on Adaptive Horizons

The determination of optimal horizon *N*_opt_ is critical in UFIR filtering and smoothing [42]. Because a reference signal is unavailable for ECG data, *N*_opt_ can be found following [55] via the mean square value (MSV) *V*(*N*, *p*) = *E*{*ε*_*n*_(*N*, *p*)^2^} of the measurement residual $\varepsilon_{n}(N,p)=s_{n}-{\hat{x}}_{n|n-p}(N)$. It has been shown in [55] that *N*_opt_(*p*) can be estimated by minimizing the derivative ∂*V*(*N*, *p*)/∂*N* as

(12) $$\begin{array}{c} {\hat{N}}_{opt}\left(\;p\;\right)=\underset{N}{arg\;min}\frac{\partial V\left(N,p\right)}{\partial N}+1. \end{array}$$

**Figure 2:**
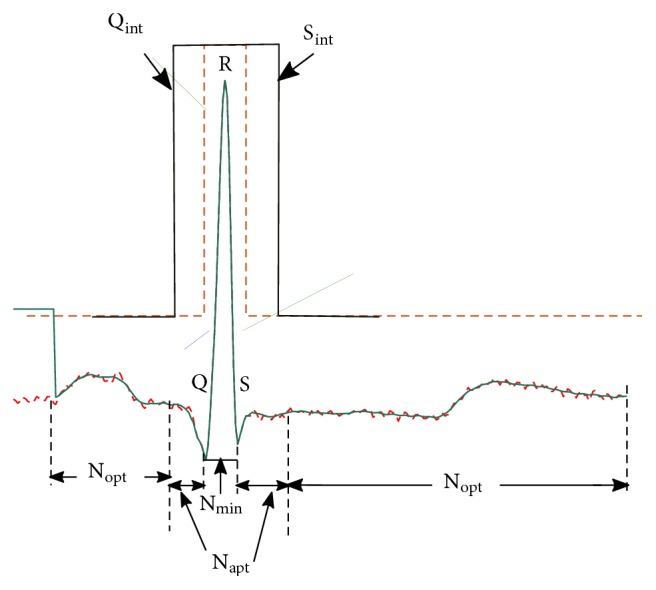
An original single ECG pulse corrupted by measurement noise (dashed) and the denoised pulse (sold). Slow parts of the ECG pulse require denoising with *N*_opt_ and a fast excursion requires a minimum horizon length of *N*_min_ = *l* + 1 points. Here, Q and S are the morphology features of ECG signal; Q_int_ and S_int_ represent the window width allowed for the adaptation. The adaptive horizon *N*_apt_ ranges from *N*_opt_ to *N*_min_. (Figure 2 is reproduced from Carlos Lastre-Dominguez et al. (2017) [42, 43], (Copyright 2017, IEEE).)

To optimize the horizon, let us consider a single ECG pulse shown in Figure 2. As can be seen, the ECG pulse is slowly changing, except for a fast excursion in the QRS region. The slow background requires an optimal horizon *N*_opt_≜*N*_opt_(*p*) in order to provide best denoising with no essential bias. On the contrary, the QRS region requires a minimum horizon *N*_min_≜*N*_min_(*p*) of *l* + 1 points to track the behaviour exactly. The horizon *N* must thus be adaptive.

#### 2.2.3. General UFIR Smoothing Algorithm

The general UFIR smoothing algorithm is represented with a pseudocode listed as Algorithm 1. It requires values of **S**_*N*_, *N*, and *l* described in Section 2.1.1. Function **CalculateG** provides a vector **G** = [1  0 ⋯ 0]^*T*^ and** CalculateV** calculates matrix **V** given by (8). Vector **B** contains the UFIR filter coefficients (6). Provided there are  **V** and **B**, the *l*-degree matrix **W**_*l*_ is computed and estimate ${\hat{x}}_{n|n-p}(N)$ is provided by (2b). We will use this algorithm at different horizons as** smoothingUFIR** function.

#### 2.2.4. Computing *N*_*opt*_ for ECG Data

Optimal horizon *N*_opt_ is provided by the algorithm designed, with a pseudocode listed as Algorithm 2. This algorithm requires the following variables: heartbeats data *s*_*i*_, filter degree *l*, a set of heartbeats *beats*, the number of heartbeats *Nbeats*, and the window width *Interval*.

By defining (8) and (9) and then analysing (12), the filter coefficients specified by (13) are obtained for given *l*, *N*, and *p*. Next, coefficients are computed for (11) and estimate (1) is provided as ${\tilde{x}}_{n|n-p}(N)$. Function** IntervalQRS** is introduced to detect Q and S via data *s*_*i*_ and a value called *Interval*, which is related to the window width.

The window covers a region including Q and S and is used as *N*_min_. Because *N*_opt_ will produce highly biased estimates around Q and S, the window is split into three parts:

(13) x~n ∣ n−pN1=x~n ∣ n−pN1:Qint−1,

(14) x~n ∣ n−pN2=x~n ∣ n−pNminQint:Sint,

(15) x~n ∣ n−pN3=x~n ∣ n−pNQint:T,

where points Q_int_ and S_int_ determinate the window width for *Inteval*. The horizon *N*_opt_ is applied to the first part (13) and third part (15). In the second part (14), estimation is provided with *N*_min_.

Function** Cat** is used to concatenate estimates (13)–(15) and compute the final estimate ${\hat{x}}_{n|n-p}(N)$. Provided we have  ${\hat{x}}_{n|n-p}(N)$, function *V*_*n*_(*N*) is calculated for *s*_*i*_ in the *N* scale. This variable is saved as *V*_*i*_(*N*) to represent a whole set of data *V*_*n*_(*N*) for different heartbeats. Provided there are various values of MSV for each *s*_*i*_, an average of *V*_*i*_(*N*) is computed as *V*_avg_. Because *V*_avg_ is accompanied with ripples causing ambiguities, it is further approximated with a cubic polynomial using function** CubicFit**. The derivative applied to smoothed *V*_avg_ while solving the optimization problem (12) yields *N*_opt_.

#### 2.2.5. Denoising Algorithm for ECG Signals

 Provided there are *N*_min_ and *N*_opt_, the UFIR smoothing algorithm can be designed for ECG signals with a pseudocode listed as Algorithm 3. In this algorithm, function** smoothingUFIR** is applied to different ECG signals parts with different horizons.

Five parts of the ECG signal are recognized by function** smoothingUFIR** over points Q_int_, S_int_, S, and Q:

(16) x~n ∣ n−pN1=x~n ∣ n−pNopt1:Qint−1,

(17) x~n ∣ n−pN2=x~n ∣ n−pNaptQint:Q−1,

(18) x~n ∣ n−pN3=x~n ∣ n−pNminQ:S,

(19) x~n ∣ n−pN4=x~n ∣ n−pNaptS+1:Sint,

(20) x~n ∣ n−pN5=x~n ∣ n−pNoptSint+1:T.

In Figure 2, the first and fifth parts are defined by (16) and (20), respectively, to apply *N*_opt_. The third part represents an estimate, which is equal to the original ECG signal without noise reduction on [Q, S]. The adaptive horizon *N*_apt_ is applied to (17) and (19). Here, *N* is decreased from *N*_opt_ to with a one-time step in the QRS complex region. Beyond the QRS complex, *N* is gradually increased from *N*_min_ to *N*_opt_ with a one-time step. Finally, function** Cat** provides the ECG signal estimate at the last fifth part.

#### 2.2.6. UFIR-Based Algorithm for Features Extraction

 Provided there is denoising by Algorithm 3, in this section we develop an efficient computation algorithm for ECG signal features extraction. To this end, we first localize special points on the ECG heartbeat pulse and then compute relevant amplitudes, durations, and an angle. Unlike the approaches developed in [15, 56, 57], this algorithm is based on the *p*-shift and *l*-order UFIR smoothing filter exploited with *l* = 2 and *p* < 0. It was found out for data used that *N*_opt_ = 21 suites smooth parts of the discrete ECG signal and *N*_min_ = 3 fits the QRS complex. Note that *N*_opt_ and *N*_min_ must be specified for each of the measured ECG signals.

**Figure 3:**
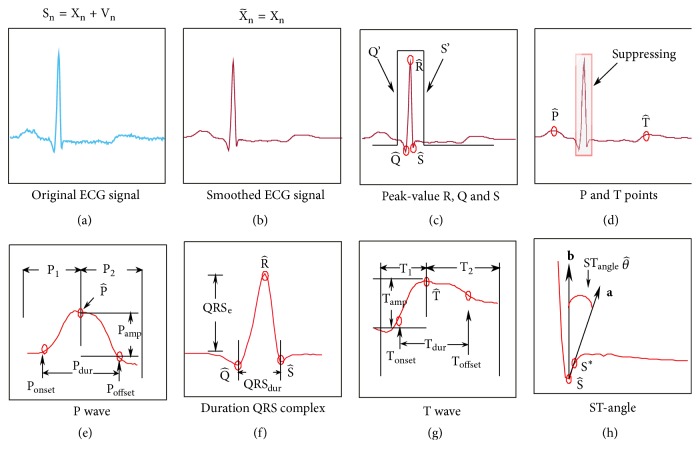
Step-by-step events representing the strategy of the ECG signal denoising and features extraction: (a) original ECG signal, (b) smoothed ECG signal, (c) peak-value R, Q, and S, (d) P and T points, (e) P wave, (f) duration of QRS complex, (g) T wave, and (h) ST-angle.

Step-by-step events representing the strategy of ECG signal denoising and features extraction are shown in Figure 3. The original discrete-time ECG signal (a) is smoothed as (b) using Algorithm 3. Then the ECG signal features are extracted as in the following:

(i) Figure 3(c): the peak value R (ECG signal maximum) is estimated as $\hat{R}$ and a window is introduced with two points, Q′ and S′. The estimate $\hat{Q}$ of Q is found as the least in the interval between Q′ and $\hat{R}$. The estimate $\hat{S}$ of S is found as the least between $\hat{R}$ and S′.
(ii) Figure 3(d): provided there are $\hat{Q}$, $\hat{R}$, and $\hat{S}$, the QRS complex is suppressed to save only P and T waves. Then the estimates $\hat{P}$ of P and $\hat{T}$ of T are obtained similarly by suppressing one of the waves.
(iii) Figure 3(e): provided there is $\hat{P}$, the P wave is split into two segments, P_1_ and P_2_, where P_1_ is extended from the initial point to P_2_. In segment P_1_, we apply the derivative. Next, we consider a small section of the resulting signal and find a global maximum. We consider it as a start point of P wave and call it P_onset_. In segment P_2_, we also apply the derivative, consider a small portion of the resulting signal, and find a global minimum. This minimum, which corresponds to the end of P wave, is called P_offset_. Values of P_onset_ and P_offset_ are located at points (Although P_p_^on^ and P_p_^off^ are omitted in Figure 3, their values represent the temporal line in the ECG signal. These points can be used to compute features of the duration and applied to R_p_, Q_p_  S_p_, P_p_, T_p_, T_p_^on^, T_p_^off^, and S_p_^*∗*^, which are described in Algorithm 4.) P_p_^on^ and P_p_^off^, respectively. Then, the duration of P wave is computed as P_dur_ = P_p_^off^ − P_p_^on^. A distance between $\hat{P}$ and the baseline is calculated and called the wave amplitude.
(iv) Figure 3(f): the QRS complex duration is obtained by the distance between points $\hat{Q}$ and $\hat{S}$. The QRS complex amplitude is provided by a distance between the baseline and $\hat{R}$.
(v) Figure 3(g): similarly, points T_onset_ and T_offset_ are obtained for the T wave by splitting this wave into two segments, T_1_ and T_2_.
(vi) Figure 3(h): the ST-angle *θ* is computed by
  (21) $$\begin{array}{c} \frac{a.b}{\left|a\right|\left|b\right|}=\cos\theta , \end{array}$$
where **a** and **b** are vectors created from $\hat{S}$ and S^*∗*^. These values are localized in S_p_ and S_p_^*∗*^. Vectors **a** and **b** have two components dependent on $\hat{S}$ and S^*∗*^. We consider a flat part, where S_p_ and $\hat{S}$ represent the origin zero point. We sum a temporal unity from the origin, obtain S^*∗*^ and S_p_^*∗*^, and rename S^*∗*^ as S_y_^*∗*^ and S_p_^*∗*^ as S_x_^*∗*^ from xy plane. We then compute **a** = S_x_^*∗*^ + S_y_^*∗*^ and **b** = 0_*x*_ + S_y_^*∗*^ and estimate *θ* via (18).

#### 2.2.7. Algorithm Design for Features Extraction of ECG Signals

A pseudocode of the algorithm designed to extract features of ECG signal is shown as Algorithm 4. Here, *ss*_*i*_ is the smoothed ECG signal represented as $\tilde{x}$ in Figure 3; *N*_b_ is the number of heartbeats; *Baseline* is a variable, which represents the reference line; *f*_*s*_ is the data sample frequency; *Interval* is a value, which determines the window width to cover Q and S points (Figure 3). The algorithm output consists of estimates of the ECG signal features such as $\hat{P}$ of P, P_amp_ of the P amplitude, P_dur_ of the P duration, QRS_*e*_ of the QRS amplitude, QRS_dur_ of the QRS duration, $\hat{T}$ of T, T_amp_ of the T amplitude, T_dur_ of the T duration, and $\hat{\theta }$ of the ST angle *θ*. All these features are extracted from the smoothed signal *ss*_*i*_.

The algorithm starts by computing $\hat{R}$ as the ECG signal maximum, using function** max**. Function** IntervalQRS** is applied to compute points Q′ and S′. The *Interval* variable determines the window width to cover the QRS complex and obtain $\hat{Q}$ and $\hat{S}$ as two minima between points Q′ and S′. Function** min** is used to find the above-mentioned points. The** supress** function is used to suppress the QRS complex. Function** max** is used to estimate P and T. Function** diff** is introduced to compute the derivatives in the P_1_, P_2_, T_1_, and T_2_ intervals. Functions** max** and** min** with function** diff** are used to find P_onset_, P_p_^on^, P_offset_, P_p_^off^, T_onset_  T_p_^on^, and T_offset_  T_p_^off^. Provided the above-mentioned values are considered, the duration is estimated of P and T features. Function** length** is introduced to compute the signal length. The *Baseline* variable determines the reference line for computing the amplitude features. This variable is equal to P_offset_. Function** vector** is used to provide vectors** a** and** b** based on S_p_, $\hat{S}$, S^*∗*^_p_, and S^*∗*^. Finally, function** arcos** is used to compute an angle between vectors** a** and** b**. Note that all the above introduced functions are available from the authors by request.

### 2.3. Validation

Several methods have been proposed during decades for ECG signal features extraction. Among these methods, the Linear Predict approach proposed in [25] and developed by Martis [28] has been recognized as one of most efficient. The method employs the following model:

(22) $$\begin{array}{c} \hat{\lambda }\left(\;n\;\right)=\overset{q}{\underset{i=1}{\sum }}\delta \left(\;i\;\right)\lambda \left(\;n-i\;\right), \end{array}$$

in which *λ*(*n*) is the original ECG pulse, *q* is the estimator order, and *δ*(*i*) is the linear prediction coefficient. The estimate $\hat{\lambda }(n)$ is provided as a linear weighted combination of *λ*(*n* − *i*), *i* = [1, *q*]. The residual error

(23) $$\begin{array}{c} \epsilon \left(\;n\;\right)=\lambda \left(\;n\;\right)-\hat{\lambda }\left(\;n\;\right), \end{array}$$

is considered as the ECG signal fraction, which cannot be predicted. To compare with the UFIR filter, we will assign *q* = 2 as suggested by Lin et al. [24].

The UFIR filter predicts estimates with *p* > 0 and both the prediction estimator (17) and the UFIR predictive filter (1) employ discrete linear prediction of the undergoing process via its noisy data. Even so, there are some zones in the ECG picture where linear predictors are unsuccessful in extracting ECG features. Therefore, a comparative analysis of different methods developed in [36–39] is required.

The real ECG data has unknown model and noise. A suitable metric is the concentration of error which is the difference between the estimate and measurement for different parts of ECG signals. The box plot allows giving indices related to the error dispersion and concentration. Moreover, a critical measure of denoising efficiency in any estimator is the MSE at its output. We provide the relevant study based on synthetic ECG signals generated using MATLAB. The ECG signal is contaminated by zero mean additive white Gaussian noise (AWGN) providing different SNR values.

The assessing performance for the features extraction is to analyse the concentration of the features seeing the effect of noise in the estimated features.

## 3. Results

### 3.1. Testing of Algorithms for Estimating *N*_*opt*_ and Denoising Algorithm

**Figure 4:**
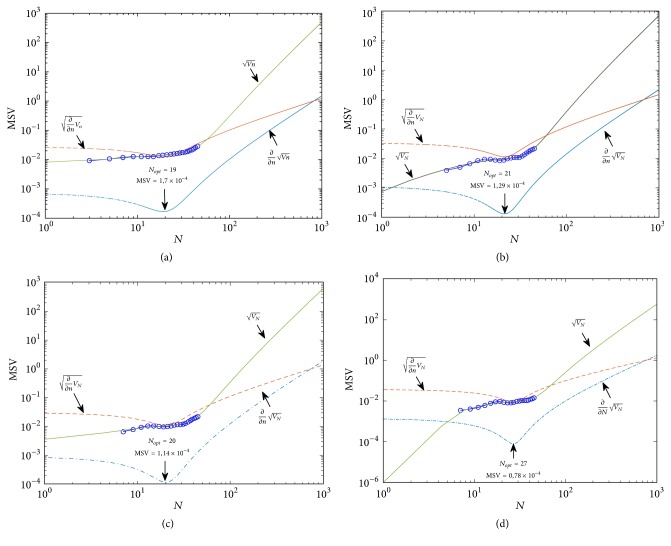
Effect of *N* on the MSV with (a) *l* = 1, (b) *l* = 2, (c) *l* = 3, and (d) *l* = 4: the MSV is circled, $\sqrt{V_{N}}$ is a cubic approximation of the MSV, and $\left(\partial /\partial N\right)\sqrt{V_{N}}$ and $\sqrt{\left(\partial /\partial N\right)V_{N}}$ are the derivatives of $\sqrt{V_{N}}$. The optimal horizon *N*_opt_ = 19 corresponds to the minimum of $\sqrt{\left(\partial /\partial N\right)V_{N}}$. (Figure 4 is reproduced from Carlos Lastre-Dominguez et al. (2017), [42], (Copyright 2017, IEEE)).

To test Algorithm 2 experimentally, we selected healthy heartbeats with 301 samples and estimated errors by allowing *N*_min_ ≤ *N* ≤ 10^3^ for *l* = 1 (Figure 4(a)), *l* = 2 (Figure 4(b)), *l* = 3 (Figure 4(c)), and *l* = 4 (Figure 4(d)).

**Figure 5:**
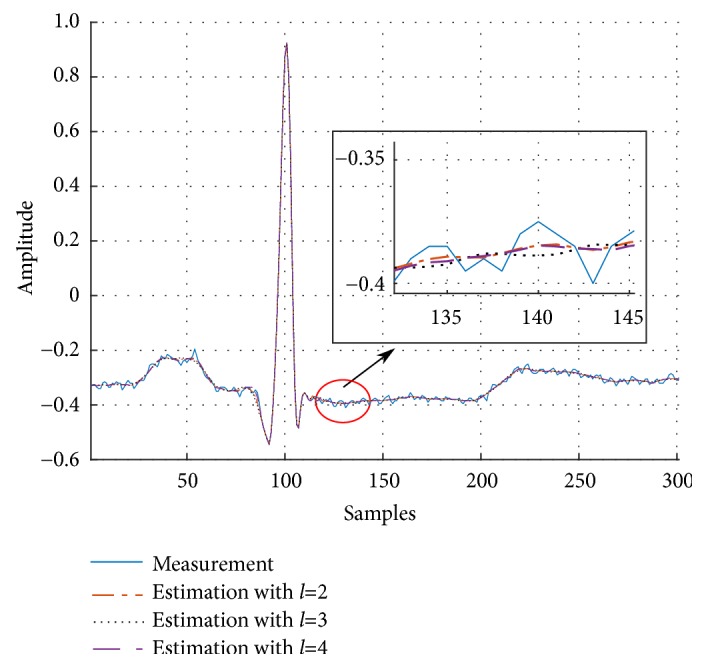
Effect of the UFIR filter degree *l* on the estimation accuracy. (Figure 5 is reproduced from Carlos Lastre-Dominguez et al. (2017), [42, 43], (Copyright 2017, IEEE)).

As can be seen, *V*_*n*_ behaves similarly for different degrees *l*. It can also be observed that *N*_opt_ generally grows with *l* and elevates to *N*_opt_ = 27 when *l* = 4. Particularly in Algorithm 3, an analysis of estimation errors produced by the 2-degree and 3-degree UFIR filters reveals no significant differences, except for the horizon length, which inherently grows with *l*. This is explained by the fact that *p* = −(*N* − 1)/2 makes the noise power gain (NPG) of both filters equal [37]. The role of *p* on the smoothing filter NPG has been studied by Shmaliy et al. in [37]. However, choosing *l* = 2 reduces the computational complexity, while saving the estimation accuracy, and we accept *l* = 2 as near optimal. Effect of *l* on the estimation accuracy is illustrated in Figure 5.

### 3.2. Critical Evaluation of Denoising Algorithms

**Figure 6:**
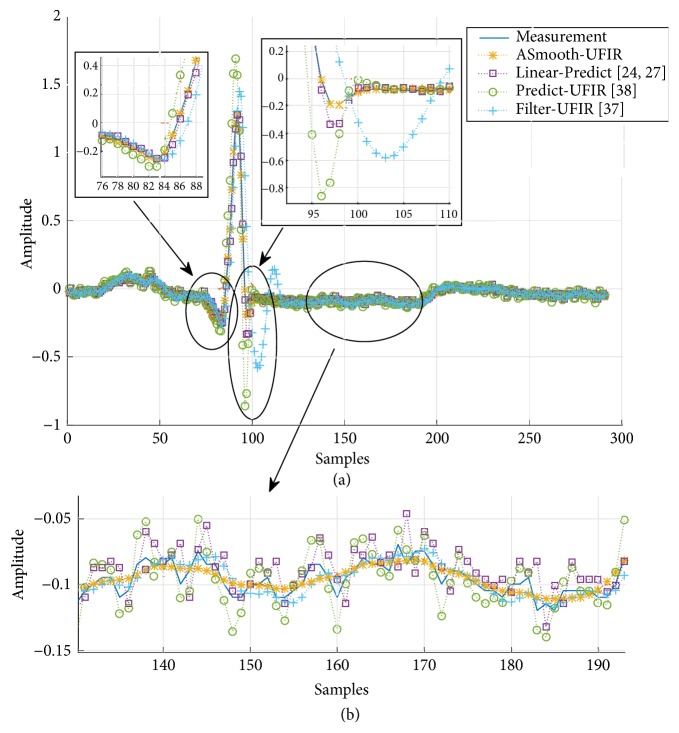
Denoising of ECG signals: (a) heartbeat estimation with different methods such as “ASmooth-UFIR" (UFIR Adaptive-Smoothing filter), “Linear Predict," “Predict-UFIR" (UFIR predictive filter), and “Filter-UFIR" (UFIR filter), and (b) segmental visualization of five estimates.

**Figure 7:**
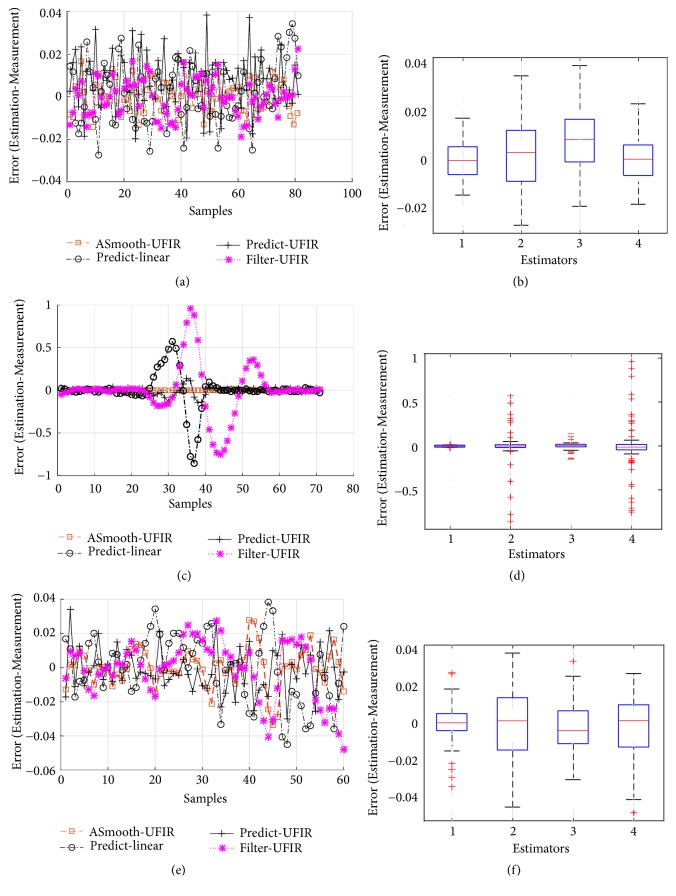
Errors produced by different estimators: (a) error in the T-wave, (b) error boxplot in the T-wave, (c) error in the QRS-complex, (d) error boxplot in the QRS-complex, (e) error in the P-wave, and (f) error boxplot in the P-wave.

In Figure 6(a), we illustrate typical denoising errors produced by the predictive filter, filter, and smoothing filter, all having batch structures. A part of the ECG signal taken from [120:200] is zoomed in Figure 6(b). The denoising errors are sketched in Figure 7.

As can be seen, all UFIR filters are successful in denoising with consistent errors. Even so, the UFIR smoothing filter does it more precisely while the predictive filter produces more errors. The medians of errors produced by the algorithms and represented with the dispersion are listed in Figure 7. This figure suggests that the UFIR smoothing filter outperforms both the UFIR filter and the standard linear predictor developed in [28] for ECG signals. An analysis of the signal-to-noise rations (SNRs) at the filters outputs will be provided next.

### 3.3. Effect of SNR on the Estimator MSE

**Figure 8:**
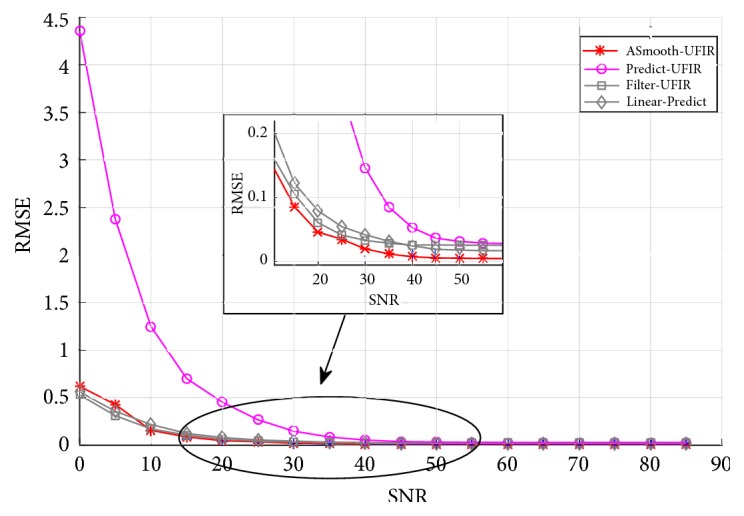
RMSEs of UFIR denoising estimators and linear predictor (Linear-Predict) as functions of SNR.

The root MSEs (RMSEs) are shown in Figure 8 as functions of the SNR depicted in decibels (dB) at 18 discrete points with a step of 5dB. It follows that the UFIR smoothing filter outperforms other solutions in a wide range of SNR values. For 0 ⩽ SNR < 15 dB, higher accuracy is achieved with a constant *N* and, for SNR > 15 dB, with an adaptive *N*.

### 3.4. Applications to ECG Signals

**Figure 9:**
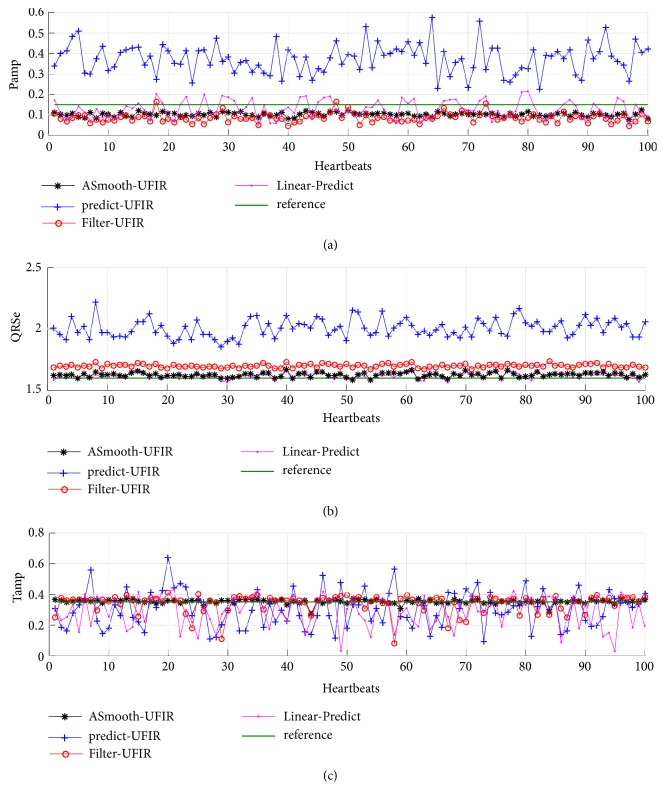
Features of the ECG signal extracted using the UFIR smoothing filter (Smooth-UFIR), basic linear predictor (Linear Predict) [25], and UFIR predictive filter (Predict UFIR): (a) P_amp_, (b) QRS_e_, and (c) T_amp_.

**Figure 10:**
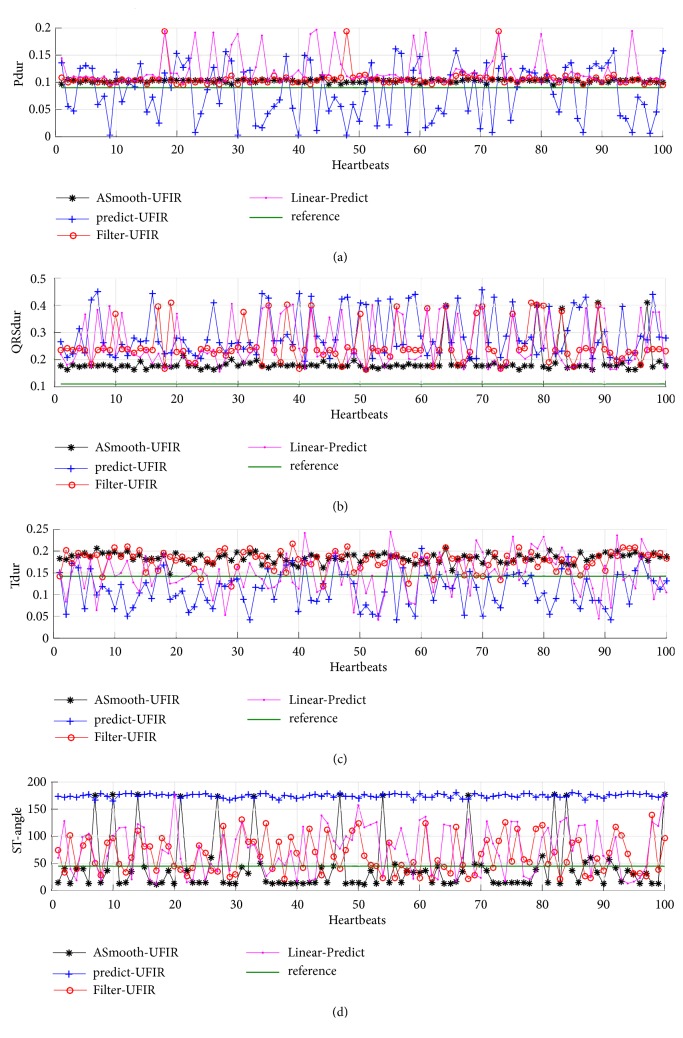
Features of the ECG signal extracted using the UFIR smoothing filter (ASmooth-UFIR), basic linear predictor (Linear Predict) [25], and UFIR predictive filter (Predict UFIR): (a) P_dur_, (b) QRS_dur_, (c) T_dur_, and (d) $ST_{angle}\;\;\hat{\theta }$. The durations are sampled with 0.0028 seconds. Also ST-angle is referenced with 45 grades.

Based upon the above developed UFIR-based approach, we now apply Algorithm 3 to the ECG signal database and extract special features depicted in Figure 1. The results obtained using the designed UFIR smoothing Algorithm 2 (UfirSmooth), UFIR predictive algorithm (Predictor UFIR), and basic linear predictor (Linear Predict) [25] are sketched in Figures 9 and 10. In these figures, 100 synthetic heartbeats are processed at each time index. This synthetic ECG signal is contaminated by AWGN at 35 dB with properties similar to the original data.

**Figure 11:**
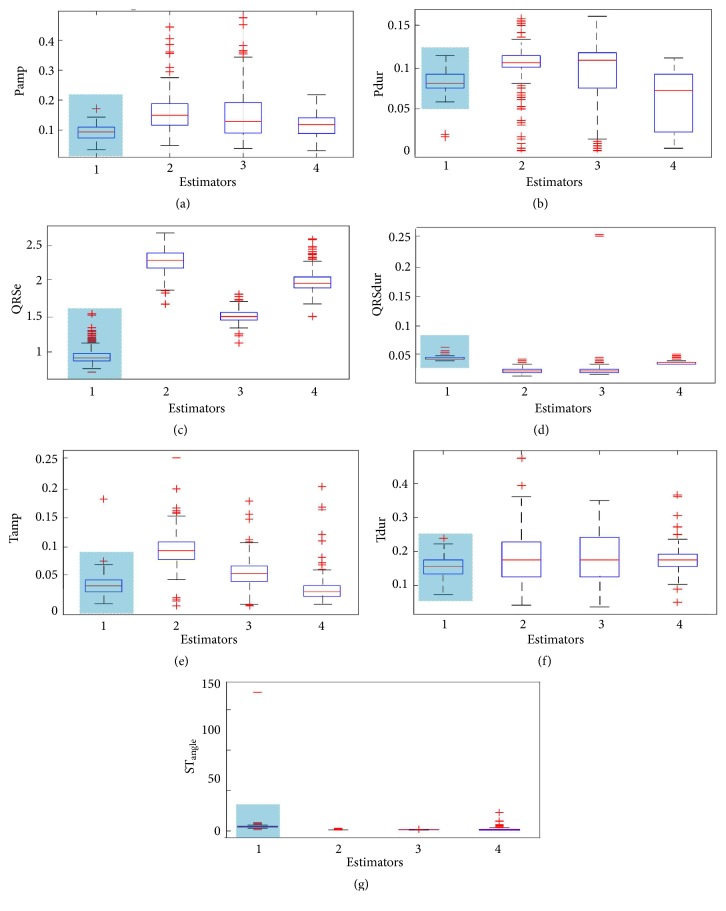
Boxplot of features of the ECG signal extracted using the UFIR adaptive-smoothing filter (Estimator 1: ASmooth-UFIR), UFIR predictive filter (Estimator 2: Predict UFIR), basic linear predictor (Estimator 3: Linear Predict), UFIR filter (Estimator 4: Filter-UFIR): (a) P_amp_, (b) P_dur_, (c) QRS_e_, (d) QRS_dur_, (e) T_amp_, (f) T_dur_, and (g) $ST_{angle}\;\;\hat{\theta }$.

In Figure 11, we show dispersions and concentrations of the estimated features about their means. Shadowed areas represent features extracted by smoothing and it follows that the outputs of the filter and linear predictor are more vulnerable. Furthermore, noise dominates in the predictive filters outputs. This experiment was based on healthy records of MIT-Arrhythmia database (lead MLII) analysing 1000 heartbeats. Overall, the UFIR smoothing approach developed in this paper always produced better estimates than other linear methods considered.

## 4. Discussion

The purpose of this study is denoising the attached noise in ECG signals using a UFIR smoothing filter for features extraction. This work is focused on the morphological features extraction individual ECG signal processing with normal rhythm. A principal finding in applying the proposed method is the considerable reduction of noise with an optimum and adaptive horizon for real ECG data. This reduction contributes determining with better precision the features associated with the heartbeat.

From analysis of errors variability in real ECG signals and SNRs based on ECG synthetic data in different estimators has shown that the UFIR smoothing filter with adaptive horizon outperforms the linear predictor [25–28] and other UFIR solutions such as the UFIR filter and UFIR predictive filter on MIT-BIH arrhythmia dataset. Let us notice again that the approaches based on linear prediction were recognized as standard for the ECG signal features extraction [28]. In this regard, better performance of the smoothing algorithm developed in this paper opens new horizons in achieving higher accuracy and reliability in detecting different kinds of heart diseases.

The UFIR smoothing filter performance was optimized by making the averaging horizon adaptive. Note that such an opportunity has not been used in the design of known linear predictors for ECG data. As a result, we have achieved the following improvements:

1. Suboptimal denoising of ECG signals with no requirements to noise, except for the zero mean assumption.
2. Unbiased filtering in the QRS region, in which the ECG signal demonstrates rapid excursions.

Such abilities of the UFIR smoothing filter have resulted in higher estimation accuracy, namely, in smaller variability of the estimated features around their mean values. In this regard, let us notice that larger variability in the standard linear predictor is due to larger errors and instability caused by unknown future data and errors in the determination of the predictor coefficients determined by the correlation method. Accordingly, errors in the determination of the prediction function lead to larger prediction errors (random and regular).

This has appeared to be particularly true for the P_amp_ and T_amp_ values, which are estimated by other methods with much larger errors. Estimates of QRS_e_ and QRS_dur_ by different methods have appeared to be consistent, because these values are not affected by noise as much as other features. Nevertheless, the UFIR smoothing filter has demonstrated smaller errors even for QRS_amp_. In the cases of both T_dur_ and angle *θ*, one watches for highly unstable estimates provided by the prediction-based filters, while the proposed UFIR smoothing filter has produced acceptable estimates. Also, it is important to clarify that the evaluation of features is analysed from the consistence of data near the average of the measurement. This is shown analysing the number of outliers. However, in this scenario, the quality features are not strictly analysed because the ECG signal used is just under normal conditions.

## 5. Conclusions

The UFIR smoothing filtering approach developed in this paper for ECG signals denoising and features extraction has demonstrated an ability to outperform the linear predictor-based one [25], which is recognized as one of the standard techniques for ECG signals. That has become possible by optimizing the order and averaging horizon for the UFIR smoothing filter in a way such that the horizon has become adaptive to different parts of ECG signals. A comparison of the UFIR predictive, filtering, and smoothing estimates has revealed a considerable difference in denoising in favor of the smoothing one. The results have also indicated that features extracted using the smoothing filter are more reliable and less prone to large deviations from average values. This is definitely an important advantage for medical needs. As a future work, we consider extracting features of ECG signals in discrete-time state-space by developing the fast iterative UFIR smoothing filtering algorithm and optimize it for different orders and kinds of heart diseases.

## Figures and Tables

**Algorithm 1:**
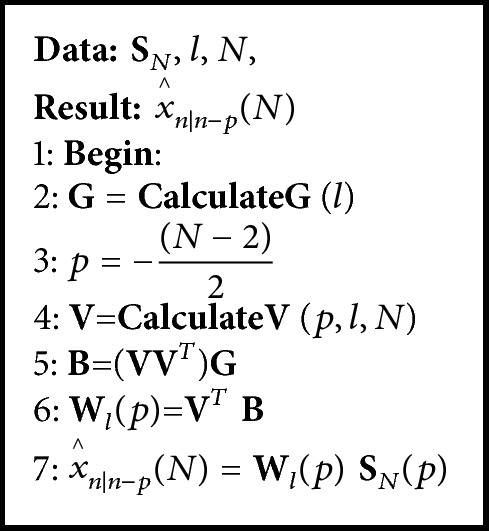
General UFIR smoothing algorithm for estimating ${\hat{x}}_{n|n-p}(N)$.

**Algorithm 2:**
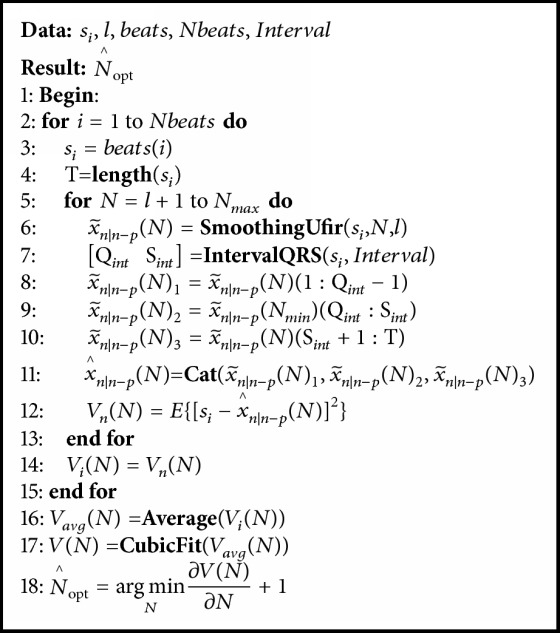
Algorithm for estimating ${\hat{N}}_{opt}$ using measurements.

**Algorithm 3:**
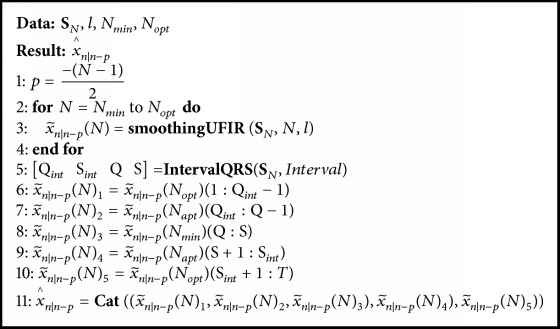
Algorithm for estimating ${\hat{x}}_{n|n-p}$ in ECG signals.

**Algorithm 4:**
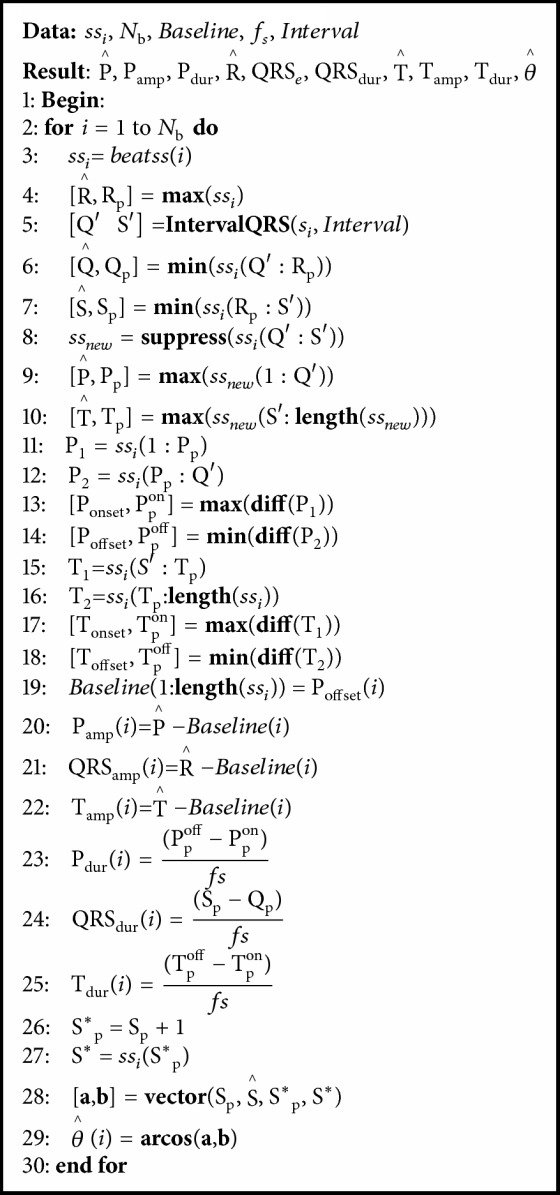
A pseudocode of the algorithm to extract morphological features of ECG signals.

## Data Availability

The data used to support the findings of this study are available from the corresponding author upon request.

## Appendix

### A. Low-Degree UFIR Functions *h*_*li*_(*N*, *p*) [37]

#### A.1. Ramp, **l** = 1

(A.1) $$\begin{array}{c} h_{1i}\left(\;p\;\right)=a_{01}\left(\;N,p\;\right)+a_{11}\left(\;N,p\;\right)i, \end{array}$$

where

(A.2) a01N,p=22N−1N−1+12pN−1+pNN2−1,

(A.3) a11N,p=6N−1+2pNN2−1.

#### A.2. Quadratic, **l** = 2

(A.4) $$\begin{array}{c} h_{2i}\left(\;p\;\right)=a_{02}\left(\;N,p\;\right)+a_{12}\left(\;N,p\;\right)i+a_{22}\left(\;N,p\;\right)i^{2}, \end{array}$$

where

(A.5) a02N,p=33N4−12N3+17N2−12N+4+12N−12N2−5N+2p+127N2−15N+7p2+120N−1p3+60p4NN2−1N2−4,

(A.6) a12N,p=−182N3−7N2+7N−2+27N2−15N+7p+30N−1p2+20p3NN2−1N2−4,

(A.7) a22N,p=30N2−3N+2+6N−1p+6p2NN2−1N2−4.
